# Quantitative linear dichroism imaging of molecular processes in living cells made simple by open software tools

**DOI:** 10.1038/s42003-021-01694-1

**Published:** 2021-02-12

**Authors:** Alexey Bondar, Olga Rybakova, Josef Melcr, Jan Dohnálek, Petro Khoroshyy, Ondřej Ticháček, Štěpán Timr, Paul Miclea, Alina Sakhi, Vendula Marková, Josef Lazar

**Affiliations:** 1 Institute of Organic Chemistry and Biochemistry, Czech Academy of Science, Praha 6, Czech Republic; 2 Center for Nanobiology and Structural Biology, Institute of Microbiology, Czech Academy of Science, Nove Hrady, Czech Republic; 3 Groningen Biomolecular Sciences and Biotechnology Institute and the Zernike Institute for Advanced Materials, University of Groningen, Groningen, Netherlands; 4 CNRS, Université de Paris, UPR 9080, Laboratoire de Biochimie Théorique, 13 rue Pierre et Marie Curie, Paris, France; 5 Institut de Biologie Physico-Chimique-Fondation Edmond de Rothschild, PSL Research University, Paris, France; 6 Faculty of Sciences, Charles University, Prague, Czech Republic

**Keywords:** Fluorescence imaging, Biological fluorescence

## Abstract

Fluorescence-detected linear dichroism microscopy allows observing various molecular processes in living cells, as well as obtaining quantitative information on orientation of fluorescent molecules associated with cellular features. Such information can provide insights into protein structure, aid in development of genetically encoded probes, and allow determinations of lipid membrane properties. However, quantitating and interpreting linear dichroism in biological systems has been laborious and unreliable. Here we present a set of open source ImageJ-based software tools that allow fast and easy linear dichroism visualization and quantitation, as well as extraction of quantitative information on molecular orientations, even in living systems. The tools were tested on model synthetic lipid vesicles and applied to a variety of biological systems, including observations of conformational changes during G-protein signaling in living cells, using fluorescent proteins. Our results show that our tools and model systems are applicable to a wide range of molecules and polarization-resolved microscopy techniques, and represent a significant step towards making polarization microscopy a mainstream tool of biological imaging.

## Abstract (web summary)

Expanding on their previous work, Bondar et al present open source software tools to reliably quantify linear dichroism and determine molecular orientations. They demonstrate the utility of the tools by imaging synthetic lipid vesicles and as well as fluorescently labelled proteins in living cells

## Introduction

Optical properties of fluorescent molecules are anisotropic. This fact has been increasingly used in biological imaging, both through anisotropy of excitation^1^ (also termed linear dichroism or photoselection) and anisotropy of emission^2^ (fluorescence polarization and fluorescence anisotropy). Anisotropy of single-photon (1P) light absorption by molecules is described by a vector, the transition dipole moment (TDM). Anisotropy of two-photon (2P) absorption is generally described by a 2P absorptivity tensor, but a simplified description by a vector analogous to the TDM is often justified^3,4^. In contrast to fluorescence polarization that typically convolves information on molecular orientation with rotational molecular diffusion and other processes, measurements of linear dichroism (LD) offer simplicity in physical interpretation: LD carries information on orientation of the absorbing molecules. Therefore, LD measurements allow observations of molecular processes that are accompanied by changes in orientation of a fluorescent moiety^5^. The presence of LD is ubiquitous^5^ in systems in which the fluorophore orientation is at least partially restricted, such as in fluorescently labeled filaments, membranes, or membrane proteins.

LD imaging, using 1P or 2P excitation, allows sensitive observations of conformational changes in membrane proteins, as well as changes in protein–protein interactions, using a single fluorescent label. It has been used for observing the organization of the nuclear pore complex^6^ and cellular microfilaments^7–9^, as well as processes such as activation of G-proteins^5,10,11^, rearrangement of septin molecules during yeast cell division^12^, changes in intracellular calcium concentration^5^, changes in cell membrane voltage^13^, and others^14^. Relying on a single fluorescent label allows observations of molecular processes under conditions closer to natural that allowed by resonance energy transfer imaging techniques. Reliance on a single label also allows multiplexing. Apart from allowing detection of molecular processes taking place, LD imaging can also provide insights into the structure of the observed proteins and protein complexes. LD imaging can be easily implemented on common laser scanning microscopes, by adding a rotatable half-wave plate^15^, a photoelastic modulator^1^, or an electro-optic modulator (Pockels cell)^5^.

Despite its advantages, LD imaging remains a domain of a few specialized laboratories. This is at least partly due to its demands for image processing and mathematical analysis, particularly when quantitative structural information is desired. Although the mathematical description has already been independently described several times^2,5,15,16^, it remains too challenging for routine use in biology. Although software that aids in processing polarization microscopy images has been developed and sometimes made publicly available^9,17^, reliable LD quantitation and interpretation in terms of molecular orientations remains a challenge. It is the goal of the present work to make LD imaging accessible to laboratories that perform mainstream biological imaging, by providing simple-to-use yet powerful software tools, along with suitable, verified experimental model systems.

## Results

### Software tools

To make LD imaging accessible, we have now streamlined and largely automated the required image processing and analysis, by developing a set of open-software tools for processing and quantitative analysis of polarization-resolved fluorescence microscopy images (https://github.com/jlazarlab/polarization_microscopy_macros)^18^. The macros facilitate visualizing and quantitating LD, as well as using LD to derive quantitative information on fluorophore orientation with respect to a lipid membrane or a filament, using a set of images acquired with two or more distinct linear polarizations of excitation light.

**Fig. 1:**
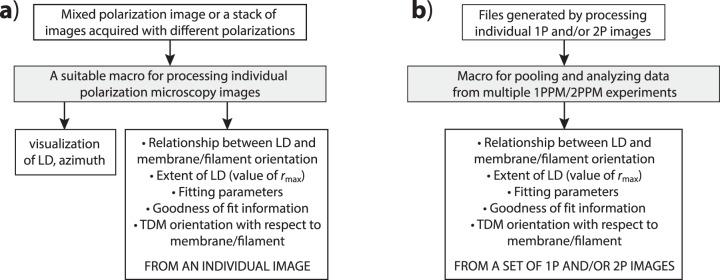
Schematic representations of the core image-processing algorithms. **a** Macros for processing individual polarization images. **b** Macro for pooling and analyzing the pooled 1P and/or 2P data.

The core of the tools comprises two ImageJ macros (Fig. 1): a macro for processing individual polarization microscopy images (Supplementary Videos 1 and 2), and a macro for pooling and analyzing data from multiple polarization microscopy experiments (Supplementary Video 3). Other tools include a macro for finding pairs of Gaussian distributions of molecular orientations that are consistent with the results of polarization microscopy experiments (Supplementary Video 4), a macro showing molecular distributions consistent with a particular combination of LD parameters, a macro for calculating LD expected for a particular (user-defined) Gaussian distribution of molecular orientations, as well as accessory macros for correcting bleaching artifacts within stacks of images acquired with distinct excitation light polarizations, generating images describing color coding of LD and azimuth information, and others.

**Fig. 2:**
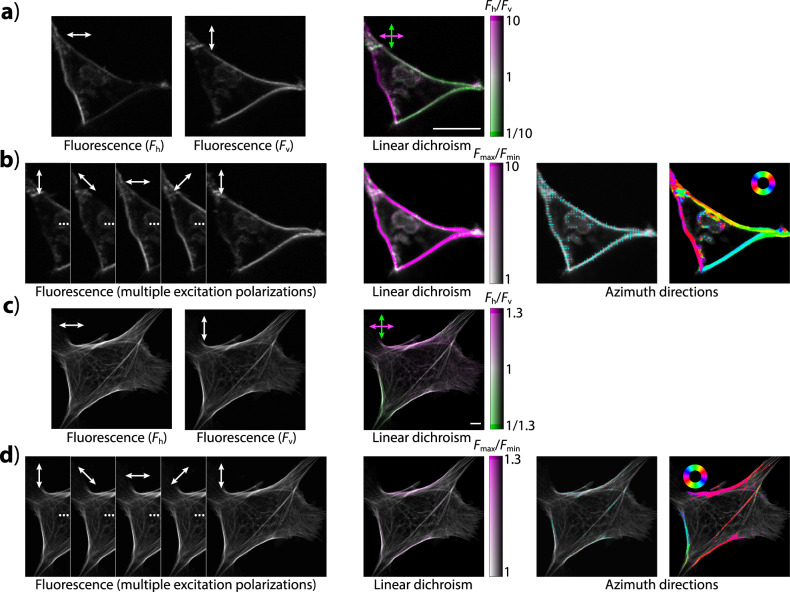
LD visualization. **a** Images of a cell expressing a membrane-localized fluorescent protein construct (dlmeGFP), observed using 2P excitation light polarized horizontally and vertically. Left: unprocessed images; Right: after processing, visualizing fluorescence intensity as brightness and the extent of LD (*r*) as hue (indicated by the color bar). **b** Images of the cell in (**a**), but acquired with multiple polarizations of the excitation light, before and after processing. Brightness indicates fluorescence intensity; hue indicates LD (*r*_max_), as shown by the color bar. Azimuth directions are shown as directed lines or by color (as shown by a color wheel). **c**, **d** Similar to (**a**), (**b**), but a cell with microfilaments stained with Texas Red/phalloidin, showing only a subtle LD. Scale bars: 20 µm.

Briefly, the extent of LD in polarization microscopy images is visualized by hue, while fluorescence intensity by brightness (Fig. 2). In images obtained using two distinct linear polarizations, LD is visualized using the dichroic ratio (*r*), defined as the ratio of fluorescence intensities excited by light polarized horizontally and vertically in the image (*r* = *F*_h_/*F*_v_). In images acquired with at least four distinct polarizations, LD is calculated by fitting the values of fluorescence intensity observed with different excitation light polarization directions (angle *θ*) by a generic cos^2^ function (*F* = *A* + *B* cos^2^(*θ* − *C*)). The ratio of maximum and minimum of fluorescence intensity (*r*_max_ = *F*_max_/*F*_min_ = (*A* + *B)*/*A*) is shown as hue. Apart from visualizing the magnitude of LD, the macro also allows visualizing the direction of polarization that yields the highest fluorescence intensity (also termed molecular “azimuth”). In case of two-polarization images, this is done by the colors indicating the extent of LD. In multiple-polarization images, molecular azimuths are shown as an image overlay, consisting of lines of a selected spacing, length, and color. By setting the line spacing interval to 1 pixel, the line length to a small value, and the color to indicate azimuth direction, azimuths can be visualized as colorful azimuth maps.

**Fig. 3:**
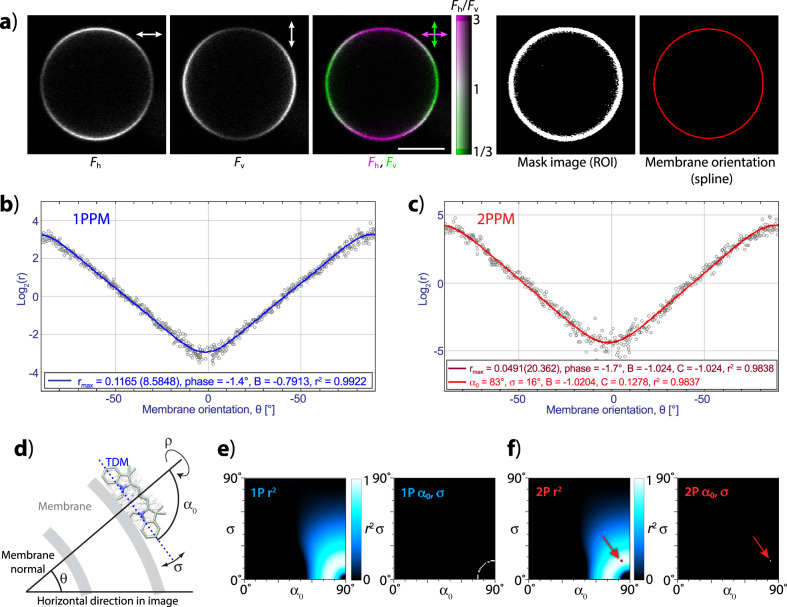
Quantitative analysis of polarization microscopy images of DiI-stained vesicles. **a** From left: a vesicle observed with 1P excitation polarized horizontally and vertically, after image processing visualizing LD, a segmentation image defining the pixels of interest, and a spline approximating the vesicle shape. Scale bar: 10 µm. **b** 1P LD (expressed as log_2_(*r*)) of the vesicle in (**a**), as a function of membrane orientation (angle *θ*). The blue line shows a fit by a parametric function describing 1P LD. **c** Same as (**b**), but for 2P excitation. The dark-red line shows a fit of the data by a generic parametric function describing 2P LD. The light-red line is a fit by a parametric function whose parameters have been restricted to those consistent with a Gaussian distribution of molecular orientations. **d** Mathematical model used for describing molecular orientation with respect to a membrane by a wrapped Gaussian distribution defined by a mean tilt angle (*α*_0_) and tilt angle distribution width (*σ*). Free rotation along the membrane normal (angle *ρ*) is assumed. **e** Agreement of the 1P LD data shown in (**b**) with different combinations of *α*_0_ and *σ*. Left: heat-map of goodness of fit (coefficient of determination, *r*^2^); right: *α*_0_, *σ* combinations consistent with the data. **f** Same as (**e**), but for 2P LD data shown in (**c**). The best fit *α*_0_, *σ* combination is indicated by an arrow.

Apart from visualizing LD, our algorithms also allow quantitating it (Fig. 3 and Supplementary Data 1). In order to perform LD quantitation, the macros aid the user in segmenting the image and approximating the shape of the investigated feature by a spline. The procedure yields a plot of LD (quantitated as *r* or log_2_(*r*)) as a function of the direction of excitation light polarization with respect to the observed feature (angle *θ*, as shown in Fig. 3d). By fitting the plotted data by a parametric function, the algorithm obtains the value of *r*_max_ (a value of LD characteristic of the observed system; in two-polarization observations equal to *F*_h_/*F*_v_ for *θ* = 0), information on data quality, as well as values of fitting parameters (*B*_1P_ for 1P data; *B*_2P_ and *C*_2P_ for 2P data) that can be used to interpret the observed LD in terms of molecular orientations.

To interpret LD in terms of distributions of molecular orientation, the macros describe molecular orientations by a wrapped Gaussian distribution, characterized by a mean tilt angle (*α*_0_) and a tilt angle distribution width (*σ*) (as shown in Fig. 3d). While data from 1P polarization microscopy observations allow identifying only a range of Gaussian distributions that agree with the LD data (Fig. 3e), 2P polarization microscopy allows finding a single Gaussian distribution of molecular orientations that matches the experimental data best (Fig. 3f). The results of this interpretation are summarized in a multislice heat map image showing agreement (quantitated by the coefficient of determination *r*^2^, RMSD, and chi-squared) between the LD data and the values expected for different wrapped Gaussian distributions (defined by parameters *α*_0_ and *σ*) of molecular orientations. For 2PPM images, the macro also finds a single combination of *α*_0_, σ that matches the 2PPM data best.

Testing with nonliving systems.

In order to test the performance of the software tools at LD quantitation and interpretation, we applied them to the previously studied^3,16,19^ system of giant unilamellar vesicles (GUVs) containing the lipophilic fluorescent dye DiI, imaged by the techniques of 1PPM and 2PPM. In 1PPM and 2PPM, the direction of polarization of the excitation light is alternated between the acquisition of individual pixels (or pixel rows) within an image. When processing images acquired by 1PPM or 2PPM, the macro for processing individual mixed polarization images (Supplementary Fig. 2 and Supplementary Video 1) deconvolves the mixed polarization image into two images, each containing information on fluorescence intensity obtained with one polarization of the excitation light. The macro then combines the information from these two images, and generates an image visualizing linear dichroism (Fig. 3).

**Fig. 4:**
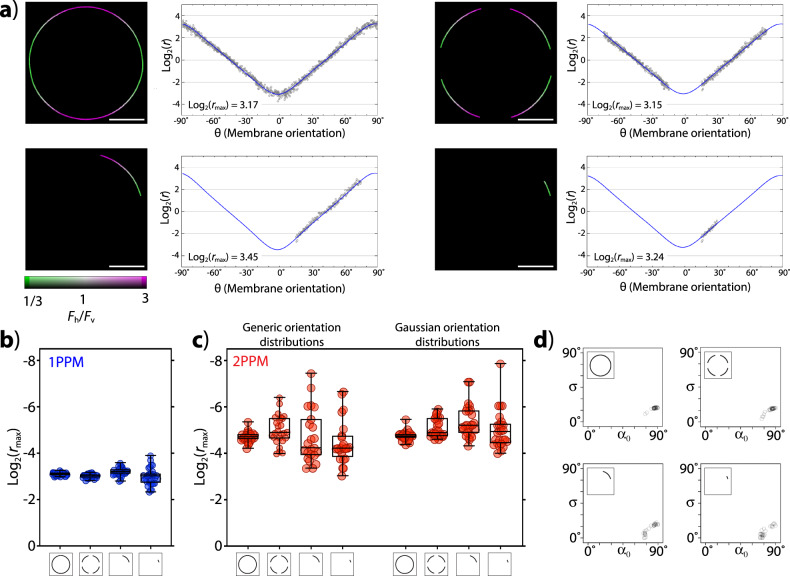
Obtaining quantitative information on LD from limited data, illustrated on DiI-stained GUVs. **a** Left panels: 1PPM images of GUVs or sections of GUVs used for analyses, colored according to LD as indicated by the color bar. Scale bars: 10 µm. Right panels: graphs of the corresponding dichroic ratio data (expressed as log_2_(*r*)), as a function of membrane orientation (angle *θ*), with the value of log_2_(*r*_max_) shown. The data fit used to obtain the value of log_2_(*r*_max_) is shown in blue. **b** Values of log_2_(*r*_max_) derived from 1PPM data obtained from images of full vesicles or their sections (as indicated). Median values, 25^th^ and 75^th^ percentiles, and ranges are shown. **c** Values of log_2_(*r*_max_) derived from 2PPM data obtained from images of full vesicles or their sections (as indicated). Left side: fitting with nonrestricted values of *B*_*2P*_ and *C*_*2P*_. Right side: fitting using values of *B*_*2P*_ and *C*_*2P*_ consistent with a Gaussian distribution of molecular orientations. Median values, 25^th^ and 75^th^ percentiles, and ranges are shown. **d** Values of *α*_0_, *σ*, derived from images of full vesicles or their sections (as indicated by the insets).

Our 1PPM and 2PPM observations of DiI-stained GUVs (Fig. 4 and Supplementary Data 1) yielded values (Supplementary Table 1) of log_2_(*r*_max_) and Gaussian distribution parameters similar to those we previously determined by polarization microscopy and verified by molecular dynamics simulations^3^. Importantly, our tools can yield useful information even from limited imaging data (Fig. 4 and Supplementary Data 1). With 1PPM, reliable values of log_2_(*r*_max_) can be obtained even from very small segments of vesicle outlines. Although 2PPM images also allow obtaining accurate log_2_(*r*_max_) values from very small sections of vesicle outline, the variance of log_2_(*r*_max_) values quickly increases with the decreasing portion of vesicle outline used for data analysis. The large variance of 2PPM data from small sections of vesicles limits the accuracy of determinations of molecular orientations (values of *α*_0_, *σ*) (Fig. 4d).

**Fig. 5:**
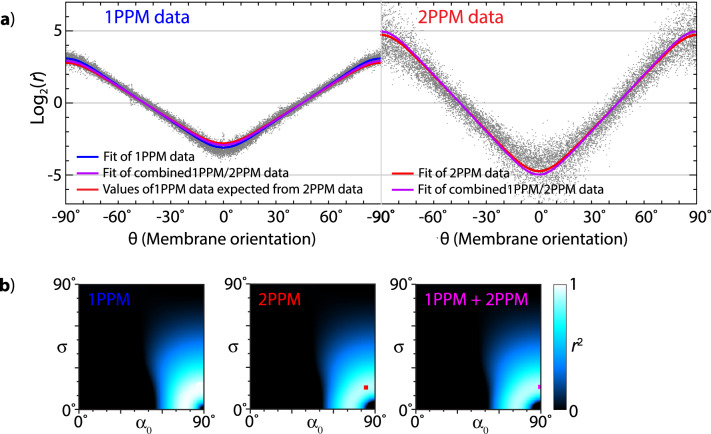
Combining single- and two-photon data, illustrated on DiI-stained GUVs imaged by 1PPM and 2PPM. **a** Fitting of combined 1PPM and 2PPM data from multiple images. 1PPM data are shown in the left part of the plot; 2PPM data are shown in the right part of the plot. Shown in blue: best fit of 1PPM data. Shown in red: best fit of 2PPM data. Since two-photon data allow predicting single-photon data, a prediction of 1PPM data based on the 2PPM fit is also shown. Shown in magenta: best fit of the combined 1PPM and 2PPM data. **b** Heat maps of goodness of fit (expressed as *r*^2^) of 1PPM data (left), 2PPM data (middle), and combined 1PPM/2PPM data (right) for a range of distributions of molecular orientations (described by values of *α*_0_, *σ*).

In order to improve the accuracy of *α*_0_, *σ* determinations, information from single- and two-photon polarization microscopy experiments can be combined (Fig. 5 and Supplementary Data 1). This can be accomplished by the macro “Combine 1P, 2P data” (Supplementary Video 3). The macro pools the log_2_(*r*(*θ*)) data from single- and two-photon experiments (Supplementary Data 1) and performs fitting on the combined data set. When applied to our DiI GUV results, the combined 1PPM and 2PPM data suggest a good, but not a perfect fit of the DiI molecular orientations by a wrapped Gaussian distribution. Another, simpler but less informative way to combine information from various experiments is by using the values of log_2_(*r*_max_) or *r*_max_ and the accessory macro “Determine alpha0, sigma from *r*_max_, log2(*r*_max_)”.

In order to characterize more complex distributions, we developed a procedure (macro “Fitting by two Gaussian distributions”, Supplementary Video 4) that searches for a combination of two Gaussian distributions of molecular orientations that would match the values of parameters *B*_*1P*_, *B*_*2P*_, and *C*_*2P*_ obtained from the single- and two-photon polarization microscopy data. Applying this procedure to the values of the *B*_*1P*_, *B*_*2P*_, and *C*_*2P*_ parameters derived from our DiI 1PPM and 2PPM data yields a number of combinations of wrapped Gaussian distributions that fit the data well (Supplementary Table 2). Their shared feature is a dominant component with *α*_0_ close to 90° (TDM in the plane of the lipid membrane) and *σ* around 10°, consistent with our previous results^3^. However, the differences in goodness of fit between various pairs of wrapped Gaussian distributions are too small to make meaningful distinctions.

### Observations of model FP-containing membrane-attached constructs in living round cells

**Fig. 6:**
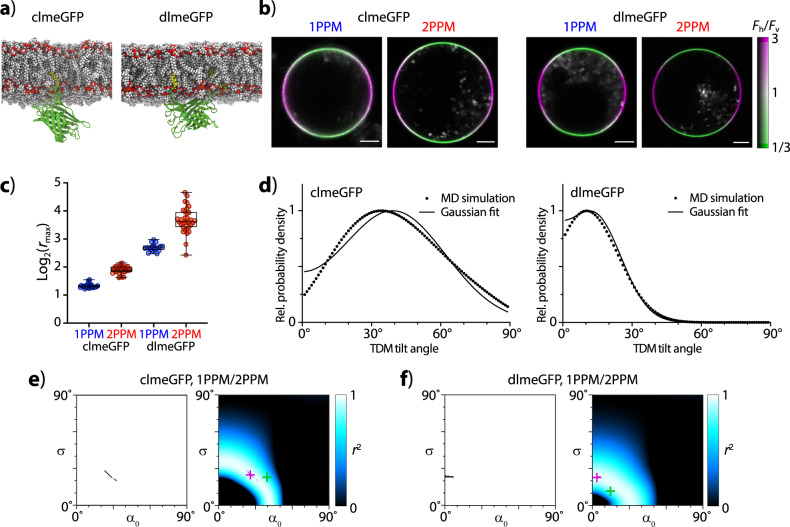
Deriving quantitative information on molecular orientations from images of model fluorescent protein constructs (clmeGFP and dlmeGFP) in round cells. **a** Renderings of the construct structures from MD simulations. **b** Images of round cells expressing clmeGFP (left) and dlmeGFP (right), obtained by 1PPM (left panels) and 2PPM (right panels). Scale bars: 10 µm. The images are colored according to LD, as indicated by the color bar. **c** Values of log_2_(*r*_max_) derived from cells expressing clmeGFP (left) and dlmeGFP (right). Median values, 25^th^ and 75^th^ percentiles, and ranges are shown. **d** Distributions of TDM tilt angles derived from MD simulations (dots) and the corresponding best-fit wrapped Gaussian distributions (curves) for clmeGFP (left) and dlmeGFP (right). **e** Combinations of *α*_0_, *σ* values consistent with pooled 1PPM and 2PPM observations of cells expressing clmeGFP. Left: estimate of 95% confidence interval obtained by bootstrapping. Right: heat map of goodness of fit (expressed as *r*^2^) of combined 1PPM/2PPM data, for a range of distributions of molecular orientations (described by values of *α*_0_, *σ*). The *α*_0_, *σ* combination in best agreement with microscopy data is indicated by a magenta cross, with the 95% confidence area marked in magenta. The *α*_0_, *σ* combination obtained from MD simulations is indicated by a green cross. **f** Same as in (**e**), but for dlmeGFP.

To test the ability of our tools to determine molecular orientations in living systems and genetically encoded fluorescent molecules, we investigated two model FP-containing constructs (Fig. 6, Supplementary Fig. 4, Supplementary Table 1, and Supplementary Data 1). The constructs consisted of the enhanced green fluorescent protein (eGFP), made monomeric by targeted point mutations^20^, and decorated by one (clmeGFP) or two (dlmeGFP) lipid tags anchoring the constructs to the cell membrane^5,21^. In order to suppress the effects of cellular morphology and membrane wrinkling, we made cells reversibly spherical by hypotonic treatment^22^. Our experiments with different hypotonicities identified 1/10x DMEM as an optimum condition for creating round, yet still viable cells (Supplementary Fig. 3). In such cells, observed by 1PPM and 2PPM, both clmeGFP and dlmeGFP exhibited pronounced LD, with the LD of dlmeGFP being higher than that of clmeGFP. In clmeGFP, the combined 1PPM and 2PPM data (Supplementary Fig. 4) were best matched by a wrapped Gaussian distribution of molecular orientations characterized by values of *α*_0_, *σ* approximately equal to 25° (Fig. 6e). In dlmeGFP, the data were most consistent with a relatively narrow distribution of TDM orientations close to perpendicular to the membrane (Fig. 6e and Supplementary Fig. 4). Importantly, the molecular orientations derived from polarization microscopy data are in good agreement with orientations identified by molecular dynamics simulations (Fig. 6d, e), validating our experimental approach.

### Observations of FP-labeled G-proteins in living round cells

**Fig. 7:**
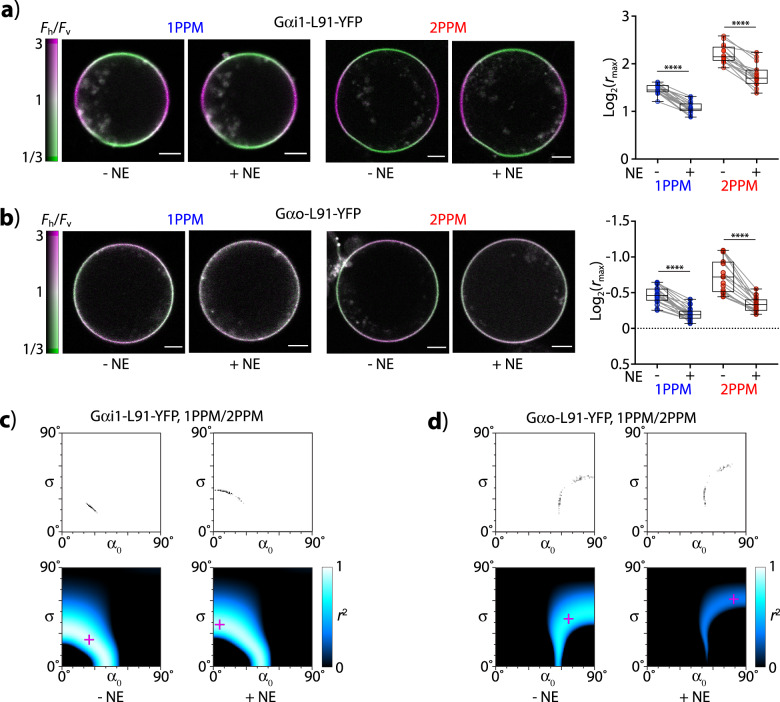
Changes in orientation of fluorescently labeled G-protein constructs during activation of the G-protein signaling pathway. **a** LD of round cells expressing G*α*i1-L91-eYFP. Left: representative images acquired by 1PPM and 2PPM, before and after activation by norepinephrine (NE). Images are colored according to LD, as indicated by the color bar. Scale bar: 20 µm. Right: changes in LD upon activation by NE. Median values, 25th and 75th percentiles, and ranges are shown. Asterisks (****) indicate statistically significant difference, with *P* value smaller than 0.0001. **b** Same as (**a**), but for G*α*o-L91-eYFP. **c** Combinations of *α*_0_, *σ* values consistent with pooled 1PPM and 2PPM observations of cells expressing G*α*i1-L91-eYFP, before and after activation. Top: estimate of 95% confidence interval obtained by bootstrapping. Bottom: Heat-map of goodness of fit (expressed as *r*^2^) of combined 1PPM/2PPM data, for different distributions of molecular orientations (described by values of *α*_0_, *σ*). The *α*_0_, *σ* combination in best agreement with microscopy data is indicated by a magenta cross. **d** Same as (**c**), but for G*α*o-L91-eYFP.

In order to demonstrate the applicability of our tools to investigations of biological processes, we performed quantitative determinations of the orientational changes occurring in fluorescently labeled G-protein constructs (G*α*_i1_-L91-eYFP and G*α*_o_-L91-eYFP) during activation of the G-protein signaling pathway (Fig. 7, Supplementary Fig. 5, Supplementary Table 1, and Supplementary Data 1). Our data show that cells expressing the *α*_2A_-adrenergic receptor, made round by hypoosmotic treatment, respond to addition of norepinephrine (NE) by decrease in LD (observable both by 1PPM and 2PPM), consistent with activation of the corresponding G-protein signaling pathway^5,10^. In both constructs, the observed decrease in LD is accompanied by a decrease in *α*_0_, and a marked increase in the width of the orientational distribution (*σ*), implying dissociation or decrease in rigidity of the G*α*_i1_–Gβγ interaction due to a GDP-to-GTP exchange, consistent with previously published studies of these constructs^10^.

### Observations of FP-bearing membrane-attached constructs in intact living cells

**Fig. 8:**
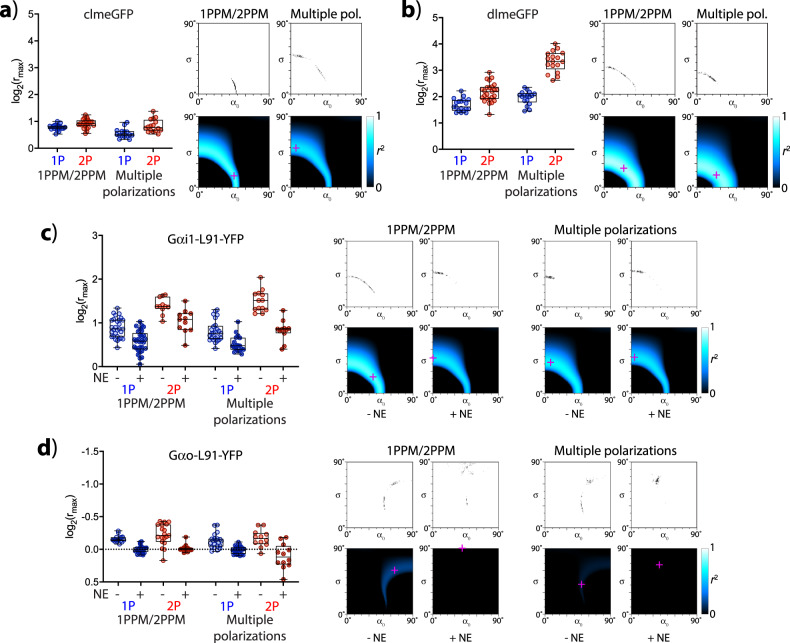
Quantitative polarization microscopy imaging of intact, living cells. **a** Cells expressing the model lipidated fluorescent protein construct clmeGFP. Left: a plot of log_2_(*r*_max_) values obtained from 1PPM, 2PPM, and from multiple polarization measurements using 1P and 2P excitation, with median values, 25^th^ and 75^th^ percentiles, and ranges shown; Right: plots showing combinations of *α*_0_, *σ* consistent with combined 1PPM/2PPM, and 1P/2P multiple-excitation polarization observations (top: 95% confidence interval estimates obtained by bootstrapping, bottom: heat-maps of goodness of fit, described by the coefficient of determination *r*^2^). The *α*_0_, *σ* combination in best agreement with microscopy data is indicated by a magenta cross. **b** Same as **a**, but for dlmeGFP. **c** Same as (**a**) and (**b**), but for G*α*i1-L91-eYFP, before and after activation by norepinephrine (NE). **d** Same as (**c**), but for G*α*o-L91-eYFP.

Next, we set out to demonstrate the applicability of our tools to images of living, intact cells. The complex shapes of their membranes can present challenges in quantitative polarization microscopy imaging. As the cells were attached, and underwent only limited movements, we were able to observe them not only by 1PPM and 2PPM, but also by using multiple polarizations of excitation light. The results of our observations of the model lipidated constructs clmeGFP and dlmeGFP, as well as the G*α*_i1_-L91-eYFP and G*α*_o_-L91-eYFP constructs (before and after activation of G-protein signaling), are summarized in Fig. 8, Supplementary Fig. 6 (cell images), Supplementary Fig. 7 (combined 1P and 2P data), Supplementary Table 1, and Supplementary Data 1. Briefly, LD in membranes of intact cells is more heterogeneous than in round cells, as reflected by values of coefficients of determination. The LD observed in intact cells (Fig. 8) is generally lower than that observed in round cells (Figs. 6 and  7). This is likely due to membrane ruffling, as well as difficulties in imaging a purely equatorial cross section of the membrane. Despite these limitations, all experimental approaches (1PPM, 2PPM, and multiple polarizations using both 1P and 2P excitation) allowed observing changes in LD associated with G-protein activation. Generally, the values of LD observed by using multiple-excitation polarizations were indistinguishable from those obtained by 1PPM/2PPM, with two exceptions: the 2P observations of dlmeGFP and of the activated form of G*α*_o_-L91-eYFP. The observed higher-than-expected LD of dlmeGFP is likely an artifact of changes to the cell shape during acquisition of the image series. The larger-than-expected variance in LD of the activated form of G*α*_o_-L91-eYFP is likely due to uncertainties in determinations of molecular azimuths in a sample showing very low LD.

Our tools permit quantitative determinations of molecular orientation (angles *α*_0_, *σ*) from LD observations of intact cells. Generally, our observations of FP-based constructs (in different systems and by different techniques) yield consistent results, with 1PPM/2PPM and multiple polarizations yielding similar results. Constructs observed in intact cells exhibit similar values of *α*_0_, but higher values of *σ* than in round cells, consistent with the effects of membrane ruffling. A notable exception is our observations of clmeGFP, where determinations of *α*_0_, *σ* from 1PPM/2PPM and multiple-polarization data diverge, although the values of log_2_(*r*_max_) obtained by the two approaches are similar. This result points to a need for high accuracy in LD measurements for the purposes of determining *α*_0_, *σ*, and shows the value of hypotonically induced round cells as an experimental model system.

### Polarization microscopy observations of fluorescently labeled filaments

**Fig. 9:**
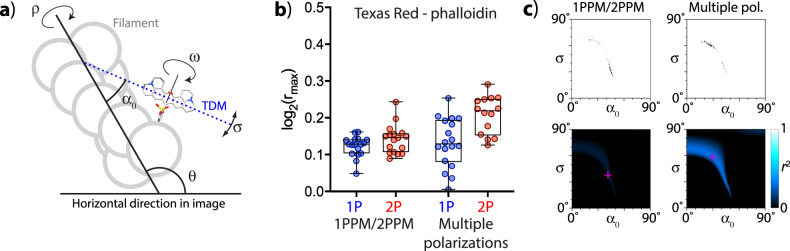
Quantitative polarization microscopy imaging of cellular microfilaments stained with Texas Red-phalloidin. **a** Model of a filamentous structure allowing describing molecular orientations by a Gaussian distribution characterized by a mean tilt angle *α*_0_ and distribution width *σ*. Rotational symmetry along the filament axis (angle *ρ*) is taken into account. In contrast, rotation along a dye-attachment axis (angle *ω*) would prevent meaningful application of the Gaussian distribution model of molecular tilt angles. **b** Quantitation of LD observed by 1PPM/2PPM, and with multiple polarizations of excitation light. **c** Plots showing combinations of *α*_0_, *σ* consistent with our 1PPM/2PPM and multiple-excitation polarization observations (top: confidence intervals obtained by bootstrapping analysis, bottom: coefficients of determination *r*^2^).

In order to evaluate the applicability of our tools to structures other than membranes, we performed polarization microscopy observations of fluorescently labeled cellular microfilaments. Because of cellular abundance of microfilaments, their overlap, and movement in live cells during microscopy observations, we used fixed cells, labeled with Texas Red-phalloidin (Fig. 2c, Fig. 2d, Fig. 9, Supplementary Fig. 8, Supplementary Table 1, and Supplementary Data 1). Briefly, the filaments exhibited very weak LD with both 1P and 2P excitation, with molecular azimuths aligned with the microfilament direction. The extent of LD observed using 1PPM and 2PPM was similar to that observed by using multiple polarizations. Small differences can be attributed to subtle errors in image alignment and to bleaching corrections needed in multiple polarization experiments. As with membrane-bound fluorophores, our algorithms can interpret the observed LD in terms of a wrapped Gaussian distribution of molecular orientations (angles *α*_0_, *σ*). In contrast to membrane-bound fluorophores, where the rotational symmetry axis (the membrane normal) is oriented perpendicular to the observed feature (lipid membrane), the rotational symmetry axis in filamentous structures is parallel to the observed structure (the filament, Fig. 9a). In order to account for this, our algorithm allows selecting which model (membrane or filament) should be used for interpreting the LD data.

Processing polarization microscopy images of fluorescently labeled microfilaments yields angles *α*_0_, *σ* (Fig. 9c), characterizing a wrapped Gaussian distribution of molecular orientation. However, the observed low coefficients of determination (Fig. 9c) for the combined 1P/2P data suggest that the molecular distribution of Texas Red deviates from the Gaussian approximation. We suspect that because of the way the Texas Red dye molecule is attached to the filament, the dye molecule possesses an additional degree of freedom (angle *ω*, Fig. 9a), inconsistent with a Gaussian distribution of molecular orientations. However, irrespective of the exact nature of the dye orientational distribution, our measurements show that the distribution is wide, but not completely random. Our results then demonstrate that our tools allow quantitating and interpreting LD observed in filamentous structures.

## Discussion

In order to make visualizing, quantitating, and interpreting linear dichroism easy and accessible, we have now developed a set of software tools. The software tools are written in the macro language of the open image-processing platform ImageJ/Fiji. This allows our software to be combined with other image processing tools (for performing stack alignment, image segmentation, or other image processing), ensures compatibility with a variety of computer systems, and facilitates modifications by users. Our software tools, a detailed guide for their use, and example files, are freely available.

The tools allow processing images of a wide range of samples (ranging from lipid vesicles stained by a synthetic dye to intact living cells expressing FP-based constructs), acquired by a variety of polarization microscopy techniques. We have demonstrated applicability of the tools to images acquired by 1PPM and 2PPM, but also by using multiple polarizations of excitation light. Furthermore, the tools can be used to process polarization microscopy images acquired by other techniques, including polarized superresolution and TIRF microscopy, as long as the macro input is in the form of an image stack with a uniform increment in the polarization direction between consecutive stack slices.

Our experiments not only demonstrate applicability of the software tools, but also assess the performance, strengths, and limitations of both the tools and the various experimental systems tested. Our observations of DiI-stained vesicles allowed us to test our tools on a previously investigated, abundantly fluorescent, geometrically simple, and nonliving model system known to exhibit LD. Our results show that the tools allow accurate LD quantitation even from very small sections of lipid membranes, particularly from 1PPM observations. The system also allowed us to interpret LD observations in terms of distributions of molecular orientations. Although general parametric orientational distributions have been used for this purpose^15^, our tools rely on wrapped Gaussian distributions, which are physically plausible as well as intuitively understandable, encompass both “pencil-like” (*α*_0_ close to 0°) and “pancake-like” (*α*_0_ close to 90°) distributions^19^, and have previously been shown to approximate orientational distributions of membrane-associated molecules well^3,23^. Although our tools do allow interpretation of LD data by more complex distributions (combinations of Gaussian distributions, analogous to combinations of Legendre polynomial elements used previously^15^), applying this ability to our DiI GUV data yielded little benefit, and suggested data overfitting. Nevertheless, the capacity of our tools to interpret LD observations by a combination of wrapped Gaussian distributions opens the possibility to characterize mixtures of molecules present in two distinct states, such as G-protein molecules in an inactive and activated state, or dye molecules in distinct molecular environments within a lipid membrane.

The robustness of our DiI GUV results and their good agreement with our previous polarization microscopy and MD studies^3^ gave us confidence to investigate more complicated molecular systems. Specifically, we investigated living cells expressing FP-containing constructs, made round by a hypoosmotic treatment. In comparison with DiI-stained GUVs, round cells show frequent outline imperfections, sensitivity to high illumination intensity, and lower photon counts. However, our tools allow automatic segmentation of round objects and easy exclusion of nondesired sections of images. When processed by our tools, 1PPM and 2PPM images of round cells allowed fast and robust quantitation of LD in a variety of constructs, as well as interpreting the observed LD in terms of molecular orientations. Our experiments allowed comparing, for the first time, molecular orientations of FP-based constructs in living cells, as determined by polarization microscopy and by MD simulations. Our results show that the two approaches are in substantial agreement, with differences that can be attributed to limitations of the coarse-grained MD model and to uncertainty about orientation of the vectors characterizing 1P and 2P absorption^24^ within the eGFP molecule. The agreement between the results of MD simulations and polarization microscopy, along with our confirmation of the ability of osmotically swollen cells to support cell signaling events^22^, establishes such cells as a suitable system for quantitative determinations of molecular orientations in living systems.

While round cells represent a useful model system and their simple shapes support easy image segmentation, our tools do allow analyzing complex shapes, such as those of cell membranes of attached cells or cellular microfilaments. This is illustrated by our observations of FP-based constructs by 1PPM and 2PPM, but also by using multiple polarizations of excitation light, modulated between acquisition of individual images. Our results show that attached cells do permit reliable LD quantitation. Importantly, values of LD obtained by distinct modes of polarization modulation are generally in good agreement. Subtle differences in LD quantitation can largely be attributed to bleaching artifacts and to cell movement that occur during measurements using multiple polarizations. Differences in LD between round and attached cells are likely due to membrane ruffling, but also to difficulties to image a purely equatorial membrane cross section in attached cells (membrane curvature radius comparable to the *z*-dimension of the point-spread function of the objective lens). This points to the value of using round cells and rapid polarization modulation (between individual image pixels or pixel rows) for precise LD observations and structural determinations.

Our work brings to light the advantages and disadvantages of various polarization microscopy techniques. It highlights an important distinction between 1P and 2P excitation: the number of molecular orientation parameters they yield. Since 2P excitation yields two parameters of molecular distributions, it provides more information than 1P excitation. In contrast, although the single parameter yielded by 1P excitation provides less information on molecular orientation, the simpler mathematical expressions needed for fitting 1P data allow more accurate LD quantitation. Therefore, 1P excitation appears more suitable for quantitating LD (when determinations of molecular orientations are not needed), while 2P excitation is necessary for determining molecular orientations. The combination of the two excitation modalities combines their advantages, and allows obtaining more detailed information on molecular orientations than either technique alone. It is, however, important to note that the current tools assume identical orientation of vectors describing 1P and 2P directionality of light absorption by fluorescent molecules, which has largely been assumed^4^, but only rarely experimentally verified^3^.

Apart from excitation modality, the number of polarizations used and the means of polarization modulation also affect the information that can be gleaned from polarization microscopy. While using multiple polarizations increases the amount of data from a sample and allows easy visualization of molecular azimuths, it also prolongs image acquisition, leading to bleaching and sample movement artifacts. In contrast, techniques modulating polarization within a single image require additional image processing. However, their speed allows imaging of fast molecular processes^13^ and accurate LD quantitation in living, moving systems.

In conclusion, our tools and experimental approaches allow accurate LD quantitation in a variety of systems, including in living cells expressing FP-labeled proteins. Our interpretation of LD in terms of orientation of the FP labels yields information consistent with MD simulations and with published data, although firm conclusions will only be possible once both the 1P and 2P directional optical properties of FPs become known. Knowledge of the directional optical properties of FPs should extend our abilities from determinations of orientation with respect to the lipid membrane of the TDMs and the presumed dominant eigenvector of absorptivity tensors, to determinations of orientation with respect to the membrane of the actual FP molecules. This ability should find uses in rational, structure-guided development of genetically encoded fluorescent probes. Developments in fluorescent unnatural amino acids and their uses in living systems will allow learning details about conformation of membrane proteins, directly in living cells. Finally, using Raman labels and polarization-resolved Raman microscopy instead of fluorescent labels and fluorescence microscopy might, in the future, allow in vivo determinations of membrane protein structures. All of those developments will necessarily rely on software tools and suitable experimental systems, which we hope will grow out of those presented here.

## Methods

### Polarization microscopy

Giant unilamellar vesicles (GUVs) were prepared by electroformation from a 1:99 molar mixture of DiI and POPC in chloroform^3^. Mammalian cells (HEK293) were cultivated in Dulbecco-modified Eagle medium (DMEM) supplemented with 10% FBS and antibiotic/antimycotic solution, at 5% CO_2_ and 37 °C. Plasmids encoding G*α*_i1_-L91-eYFP and G*α*_o_-L91-eYFP were kind gifts from M. Bünemann. Constructs clmeGFP and dlmeGFP were derived from constructs encoding lipidated versions of the eGFP^5,21^, by introducing mutations preventing dimerization^20^ (A206K, L221K, and F223R). They were prepared by gene synthesis (Thermo Fisher, Inc.). Transfections were performed in 24-well plates, using the Transporter 5 reagent (Polysciences Inc., USA, 6 µL/well), and plasmid DNA (0.2 µg/well for Gβ_1_, Gγ_2_, and *α*_2A_-adrenergic receptor, 0.3 µg/well for G*α*, and 1 µg/well for clmeGFP and dlmeGFP), according to the manufacturer’s protocol. Twenty-four hours after transfection and 24 h before imaging, the cells were transferred into an eight-chamber imaging slide (μ-slides, Ibidi GmbH, Germany). Prior to imaging, DMEM was replaced by DMEM diluted in sterile distilled water (1:9). For G-protein activation, norepinephrine (Sigma-Aldrich, 1-μM final concentration) was added by pipetting directly before imaging. Fixed BPAE cells stained with Texas Red-phalloidin (FluoCells #2) were purchased from ThermoFisher.

Imaging was carried out in a manner similar to that used before^5^. All polarization microscopy experiments were performed on a customized laser-scanning confocal microscope FV1200 (Olympus, Japan), using a 40× water-immersion objective lens (UApoN340, NA1.15, Olympus, Japan). Laser beams were passed through a Glan laser-polarizing beamsplitter (GL10B, Thorlabs, Germany), and a Pockels cell (RPM-2P, Innovative Bioimaging, USA), before entering the scanner. In 1PPM and 2PPM experiments, polarization was modulated between acquisition of subsequent pixels. In experiments using multiple polarizations, polarization was rotated in 15° steps by a superachromatic half-wave plate (SAHWP05M-700, Thorlabs, Germany) mounted in a motorized rotating mount (ELL14, Thorlabs), inserted into the microscope directly below the objective lens. Pixel dwell times of 8–16 μs, and pixel spacing between 50 and 100 nm, were used throughout. Individual measurements were made on distinct cells/vesicles, usually over the course of at least three different measurement sessions.

In single-photon experiments, fluorescence was excited by 488-nm (clmeGFP, dlmeGFP), 515-nm (G*α*_i1_-L91-eYFP and G*α*_o_-L91-eYFP), or 543-nm (DiI) wavelengths. Fluorescence was separated from excitation light by a band-reflecting dichroic mirror (DM405/488, DM458/515, or DM405/488/543/635, respectively), and a diffraction grating/slit (set to 505–605, 530–630, or 565–665 nm, respectively). Fluorescence was detected by a confocal photomultiplier detector. In two-photon experiments, a MaiTai HP 1040 (Newport/SpectraPhysics, USA) tunable femtosecond laser (set to 900, 950, or 950 nm, respectively, and attenuated to 60 mW) was used for excitation. A long-pass dichroic mirror (690-nm LP) directed the fluorescence through an emission filter (BA495-540HQ for clmeGFP, dlmeGFP, G*α*_i1_-L91-eYFP, and G*α*_o_-L91-eYFP; BA575-630 for DiI) to a non-descanned photomultiplier detector.

### LD visualization and quantitative processing of individual images

All image-processing procedures were implemented using the ImageJ macro language. Individual polarization microscopy images were processed by a “Process a polarization image” macro appropriate for the given image type (mixed polarization image, stack of images acquired with distinct polarizations). Prior to applying the polarization image-processing macro, multiple-polarization stacks of images were aligned using the StackReg plugin^25^, and adjusted for bleaching.

When applied to 1PPM or 2PPM images, the algorithm deconvolves the starting mixed polarization image into two images, each containing fluorescence excited with a single linear polarization of the excitation beam (*F*_h_, *F*_v_). After subtracting background, correcting for bleed-through between polarizations, and for unequal illumination intensities, the algorithm calculates the dichroic ratio (*F*_h_/*F*_v_) for each pixel and displays it as hue, while showing fluorescence intensity as brightness. When processing stacks of images acquired with distinct polarizations, the algorithm subtracts background before fitting the temporal fluorescence intensity profiles of individual pixels by a cos^2^ function in order to ascertain values of the ratio of the maximum and minimum fluorescence intensities (*F*_max_/*F*_min_) and directions of the molecular azimuths. The algorithm visualizes the value of *F*_max_/*F*_min_ as hue, and fluorescence intensity as brightness. Azimuth directions are shown as an overlay consisting of lines of lengths either corresponding to *r*_max_, or to a single pixel, with spacing selected by the user.

For quantitative processing, the algorithm aids in segmentation and shape approximation, either by searching for circular shapes (using the Hough circle transform plugin (https://imagej.net/Hough_Circle_Transform)), or for bright outlines of an irregular shape, while allowing the user to apply any of the segmentation tools available in ImageJ. When needed, the segmentation can be adjusted manually, by erasing some of the segmented parts using the ImageJ brush tool. The segmented parts of the image are automatically fitted by a spline, allowing manual adjustments. The segmentation/shape approximation algorithm generates a segmentation mask image and a set of spline anchor point coordinates.

In order to derive parameters describing the LD data, the algorithm associates each pixel within the segmented area with a nearest point on the spline that approximates the shape of the membrane. The orientation (angle *θ*) of the spline at that point is then associated with the value of the dichroic ratio (or log_2_(*F*_h_/*F*_v_)) of the pixel being analyzed. Value pairs (*θ*, log_2_(*F*_h_/*F*_v_)) are fitted by a parametric function, chosen by the user to match the excitation modality (single- or two-photon). LD observed with single-photon excitation is fitted^2,5,15,16^ by Eq. (1)

1 $$\log _2\left( r \right) = \log _2\left( {\frac{{F_h(\theta )}}{{F_v(\theta )}}} \right) = {\mathrm{A}}_{1P} + {\mathrm{log}}_2\left( {\frac{{1 + B_{1P}\cos (2( {\theta - \varphi _1} ))}}{{1 - B_{1P}\cos (2(\theta - \varphi _2))}}} \right)$$

where *A*_*1P*_, *B*_*1P*_, *φ*_1_, and *φ*_2_ are fitting parameters. For two-photon excitation, the data are fitted by Eq. (2)

2 $$\log _2\left( r \right) = \log _2\left( {\frac{{F_h(\theta )}}{{F_v(\theta )}}} \right) = {\mathrm{A}}_{2P} + \log _2\left( {\frac{{1 + B_{2P}\cos (2( {\theta - \varphi _1} )) + C_{2P}\cos (4( {\theta - \varphi _1} ))}}{{1 - B_{2P}\cos (2( {\theta - \varphi _2} )) + C_{2P}\cos (4( {\theta - \varphi _2} ))}}} \right)$$

with fitting parameters *A*_*2P*_, *B*_*2P*_, *C*_*2P*_, *φ*_1_, and *φ*_2_. The fitting procedure yields values of the fitting parameters, as well as a value of log_2_(*r*_max_), where *r*_max_ = *F*_h(*θ* = 0°)_/*F*_v(*θ* = 0°)_. Furthermore, the algorithm identifies a wrapped Gaussian distribution of orientations that matches the experimental data best, by using tabulated values of parameters *B*_*1P*_, *B*_*2P*_, and *C*_*2P*_ pre-calculated for a wide range of combinations of mean tilt angle (*α*_0_) and tilt angle distribution width (*σ*). Goodness of fit between experimental data and various wrapped Gaussian distributions is expressed as values of r-squared, RMSD, and Chi-squared, and displayed in the form of images (heat-maps).

Where applicable, all result values are listed as mean ± 95% confidence interval. The listed sample size (*N*) is the number of different cells or giant unilamellar vesicles analyzed. Vesicles or cells with obvious irregularities were excluded from image processing. Within a particular vesicle or a cell, sections of membrane showing irregularities (bright puncta, abrupt changes in orientation) were excluded from analysis. Experimentally determined values of log_2_(*r*_max_) were tested for normality (Anderson–Darling, D’Agostino and Pearson, and Shapiro–Wilk tests), and with an exception of very small membrane sections and some attached cell measurements passed. Statistical significance of differences between activated and nonactivated cells expressing G-protein constructs was assessed by a paired *t* test for normal distributions.

### Combining data from multiple experiments

The algorithm parses data from file directories containing results of single- and two-photon experiments, and performs fitting (as described above) on the pooled data. Fitting is performed separately for single-photon data, for two-photon data, and for the combined single- and two-photon data set, yielding values of *B*_*1P*_, *B*_*2P*_, *C*_*2P*_, and log_2_(*r*_max_). Determinations of values of *α*_0_ and *σ* are also carried out, independently for single-photon data, for two-photon data, and for the combined single- and two-photon data set. Confidence intervals of the combined single- and two-photon data fit are estimated by bootstrapping statistics.

### Finding a combination of two Gaussian distributions of molecular orientations

The algorithm uses precalculated, tabulated values of parameters *B*_*1P*_, *B*_*2P*_, and *C*_*2P*_ in order to find pairs of Gaussian distributions of molecular orientations that match the experimental data. The algorithm samples various combinations of mean tilt angles and distribution widths, as well as various fractional representations of each distribution. Agreement with experimental data is evaluated in terms of goodness of fit (r-squared) and entropy.

### Molecular dynamics simulations

Molecular dynamics simulations encompassed an orthorhombic periodic box containing a model 1-palmitoyl-2-oleoylphosphatidylcholine (POPC) membrane, an FP-based construct, and solvent with neutralizing sodium counterions. Structures of the FP-based constructs were based on the published eGFP structure^26^ (pdb ID 2Y0G), modified to include a C-terminal lipidation signal peptide (in both clmeGFP and dlmeGFP) and an internal palmitoylation signal peptide^21^, and the corresponding modifications^9^. The system was described by the coarse-grained polarizable Martini model^27,28^ (Martini 2.2p). Nonstandard amino acid residues, including palmitoylated cysteines and FP chromophores, were constructed using the standard building block approach^27^ of the Martini model. Simulations were performed using the GROMACS simulation package^29^ version 2018.3 in a mixed-precision compilation (20-fs steps) coupled to a velocity-rescaling thermostat^30^ (at 320 K), and to a semi-isotropic Parrinello-Rahman barostat^31^ (atmospheric pressure). Long-range electrostatic interactions were described by reaction field^32^, using the Verlet cutoff scheme^33^, with the Coulomb/Van der Waals cutoff of 1.1 nm. Constraints were solved using the LINCS algorithm^34^. Statistical ensembles were obtained through extensive (85 μs for dlmeGFP; 100 μs for clmeGFP) bias-free, direct MD simulations. The angle *α* was calculated as the angle between the published TDM vector prediction^24^ (as reliable experimentally determined TDM directions^35^ were not yet available) and the *z*-axis of the model system coordinates representing the membrane normal. Distributions of TDM orientations were evaluated using the kernel density estimate method^33^ (SciPy Python library^36,37^), with a bandwidth of 3.0° to account for uncertainty of the TDM orientation in the coarse-grained structure of the chromophore.

### Reporting summary

Further information on research design is available in the Nature Research Reporting Summary linked to this article.

## Supplementary information

Supplementary Information

Description of Additional Supplementary Files

Supplementary Data 1

Supplementary Movie 1

Supplementary Movie 2

Supplementary Movie 3

Supplementary Movie 4

Reporting Summary

## Data Availability

All data sets shown in plots within the main figures (Figs. 3–9) are provided as Supplementary information (Supplementary Data 1). All other data are available from the corresponding author upon request.

## Code availability

Software, including source code, documentation, and example data, is available from public repositories (GitHub; https://github.com/jlazarlab/polarization_microscopy_macros), under the GNU 3.0 general public license. Version 2.0 (10.5281/zenodo.4383287) of the software was used to produce the presented data.
