# Supplementary material for: Quantitative linear dichroism imaging of molecular processes in living cells made simple by open software tools
Source: Commun Biol. 2021 Feb 12;4:189. doi: 10.1038/s42003-021-01694-1 (PMC7881160; doi:10.1038/s42003-021-01694-1)
Supplement: Supplementary file 1 — Supplementary Information [file 42003_2021_1694_MOESM1_ESM.pdf]

Supplementary materials:

Supplementary Figure 1: Schematic of the data processing by the main macros of the macro toolset

Supplementary Figure 2: Image acquisition and deconvolution in 1PPM and 2PPM

Supplementary Figure 3: LD of cells expressing clmeGFP exposed to different hypotonic treatments

Supplementary Figure 4: LD of round cells expressing clmeGFP and dlmeGFP

Supplementary Figure 5: LD of round cells expressing G-protein based constructs

Supplementary Figure 6: Polarization microscopy images of intact cells expressing membrane-localized FP-based constructs.

Supplementary Figure 7: LD of intact cells expressing membrane-localized FP-based constructs.

Supplementary Figure 8: LD of microfilaments stained with Texas Red-phalloidin.

Supplementary Table 1: Quantitative characterization of LD of the studied fluorescent molecules

Supplementary Table 2: Fitting data from LD measurements of DiI-stained GUVs by pairs of Gaussian distributions of molecular orientations

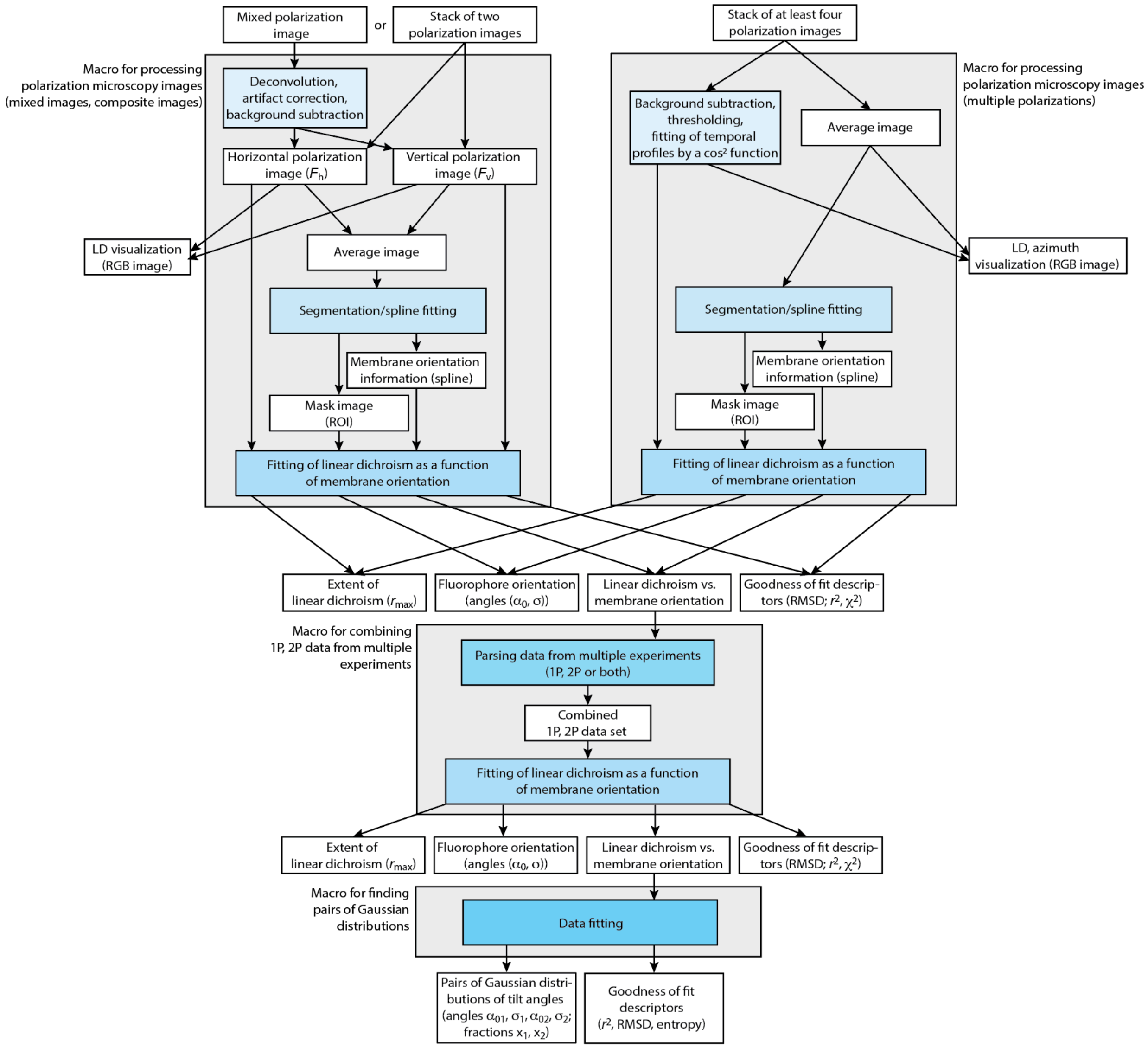

Suppl. Fig. 1: Schematic of the data processing by the main macros of the macro toolset

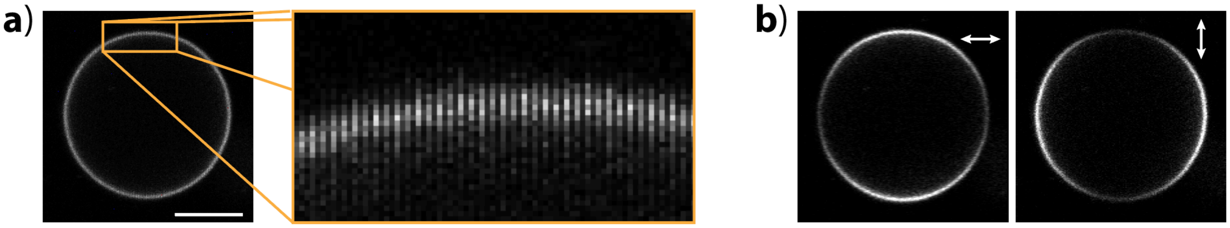

Suppl. Fig. 2: Image acquisition and deconvolution in 1PPM and 2PPM. a) An image of a POPC vesicle stained with DiI, acquired by 1PPM. Polarization was alternated between horizontal and vertical directions between acquisition of subsequent pixels, resulting in a pattern of pixel columns of alternating brightness. Scale bar: 10 µm. b) Results of deconvolution of the 1PPM image in a): a pair of images, each containing information on fluorescence intensity excited by a single linear polarization (indicated by the double-headed arrows).

`

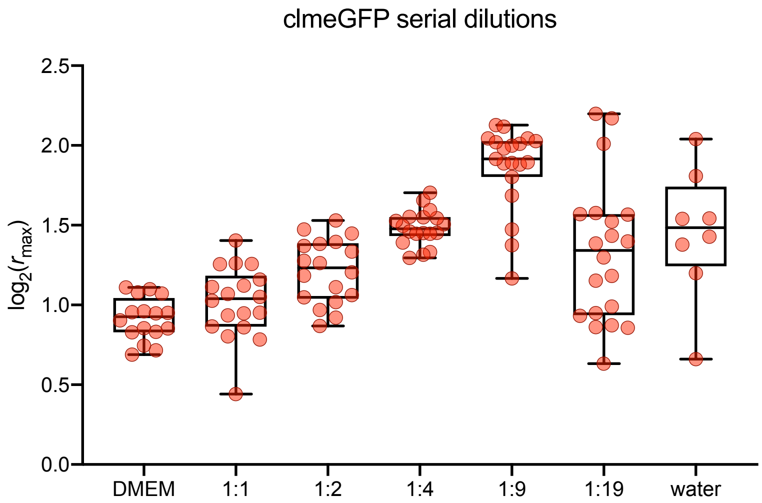

Suppl. Fig. 3: LD ($\log_2(r_{max})$) of cells expressing clmeGFP, exposed to different hypotonic treatments. Dilutions of DMEM in water, observed by 2PPM. Median values, 25th and 75th percentiles and ranges are shown.

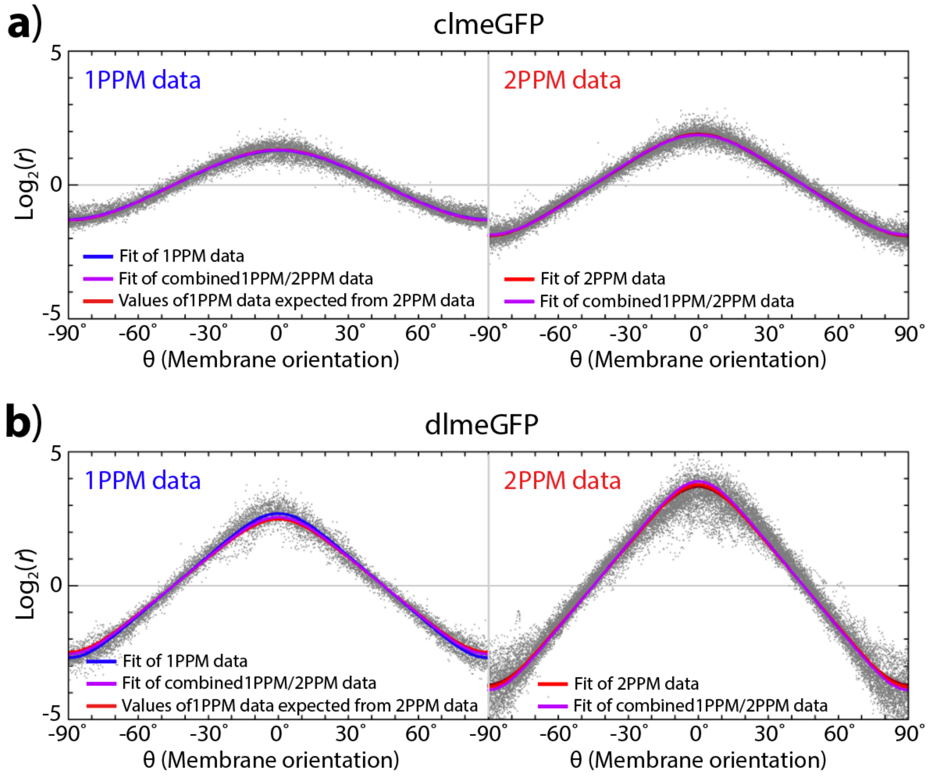

Suppl. Fig. 4: LD of round cells expressing clmeGFP and dlmeGFP. Values of $\log_2(r)$, obtained from 1PPM and 2PPM observations of multiple cells, are shown as a function of cell membrane orientation (angle θ). a) cells expressing clmeGFP; b) cells expressing dlmeGFP. Blue line – fit of 1P data; dark red line – fit of 2P data; light red line – fit of 2P data with fitting parameters restricted to Gaussian distributions of molecular orientations; magenta line – fit of combined 1P and 2P data.

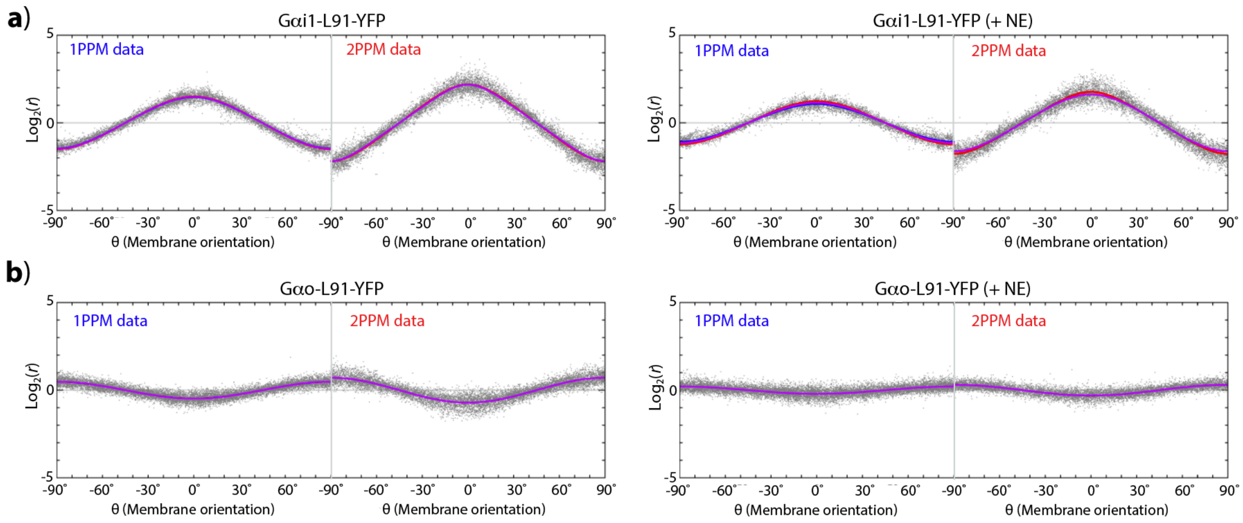

Suppl. Fig. 5: LD of round cells expressing G-protein based constructs. Values of $\log_2(r)$, obtained from 1PPM and 2PPM observations of multiple cells, are shown as a function of cell membrane orientation (angle θ). a) $G\alpha_{i1}$-L91-eYFP before activation (left) and after activation by norepinephrine (NE; right). b) $G\alpha i_o$-L91-eYFP before activation (left) and after activation by NE. Blue line – fit of 1P data; dark red line – fit of 2P data; light red line – fit of 2P data with fitting parameters restricted to Gaussian distributions of molecular orientations; magenta line – fit of combined 1P and 2P data.

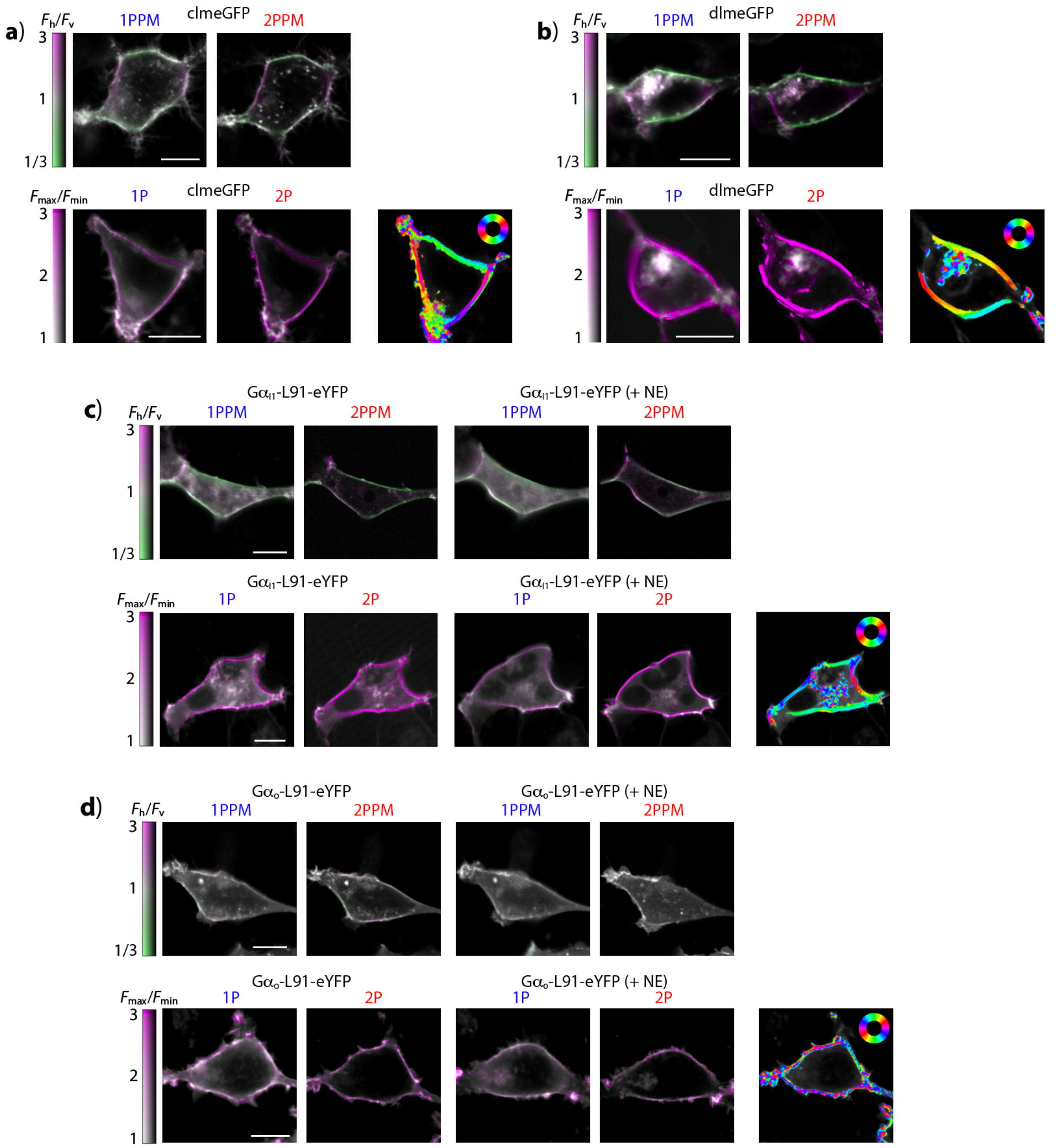

Suppl. Fig. 6: Polarization microscopy images of intact cells expressing membrane-localized FP-based constructs. Images acquired by 1PPM and 2PPM (top rows), as well as by using multiple excitation polarizations (bottom rows) are shown. Scale bars: 10 µm. a) clmeGFP; b) dlmeGFP; c) Gα_{i1}-L91-eYFP, before (left) and after activation by norepinephrine (NE; right). d) Gα_o-L91-eYFP, before (left) and after activation by NE (right).

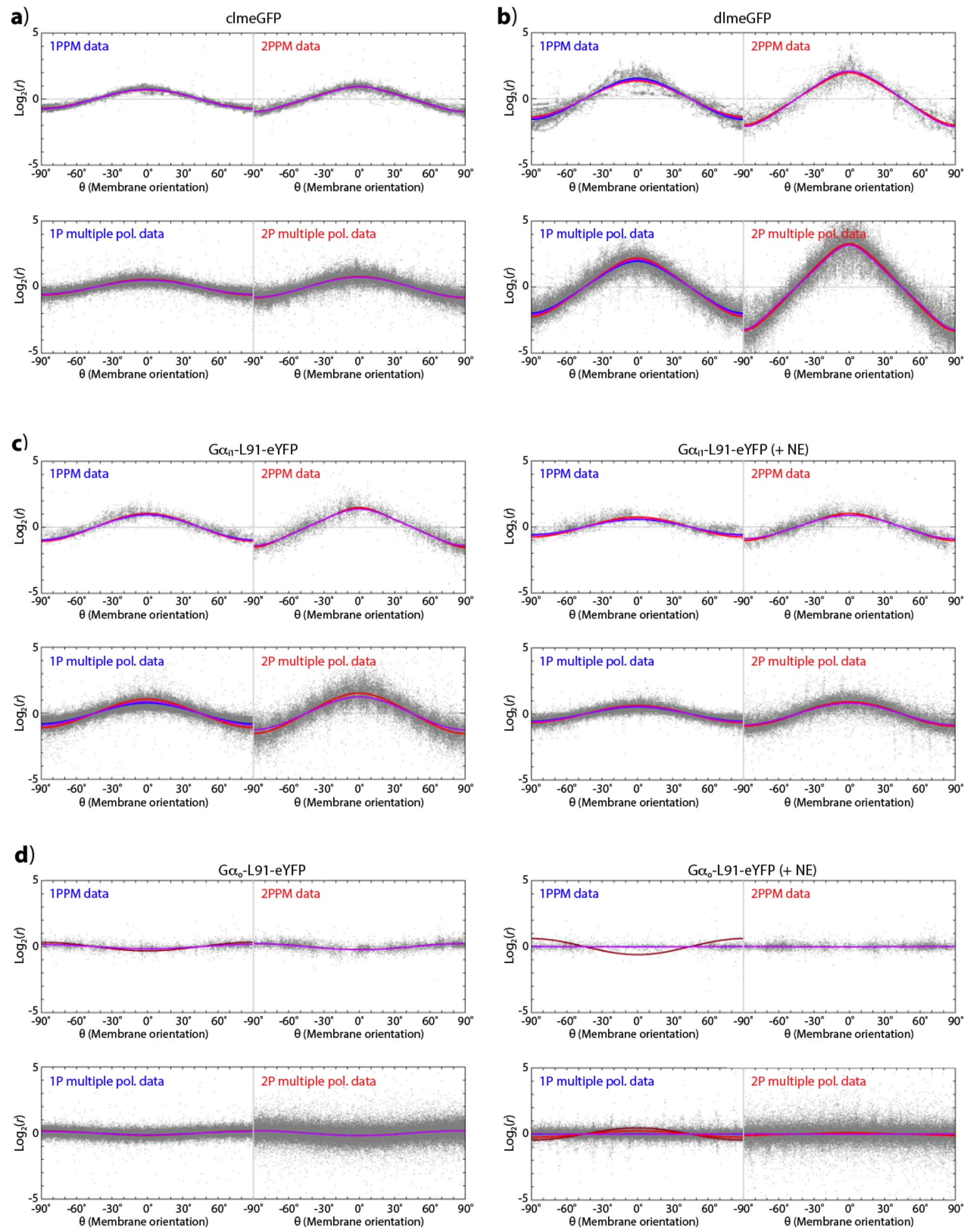

Suppl. Fig. 7: LD of intact cells expressing membrane-localized FP-based constructs. Values of $\log_2(r)$, obtained from polarization microscopy observations of multiple cells, are shown as a function of cell membrane orientation (angle θ). Upper panes show data from images acquired by 1PPM and 2PPM, bottom panes data acquired using multiple polarizations of excitation light. a) clmeGFP; b) dlmeGFP; c) $G\alpha_{i1}$-L91-eYFP before (left) and after activation by norepinephrine (NE; right); d) $G\alpha_o$-L91-eYFP before (left) and after activation by NE (right). Blue line – fit of 1P data; dark red line – fit of 2P data; light red line – fit of 2P data with fitting parameters restricted to Gaussian distributions of molecular orientations; magenta line – fit of combined 1P and 2P data.

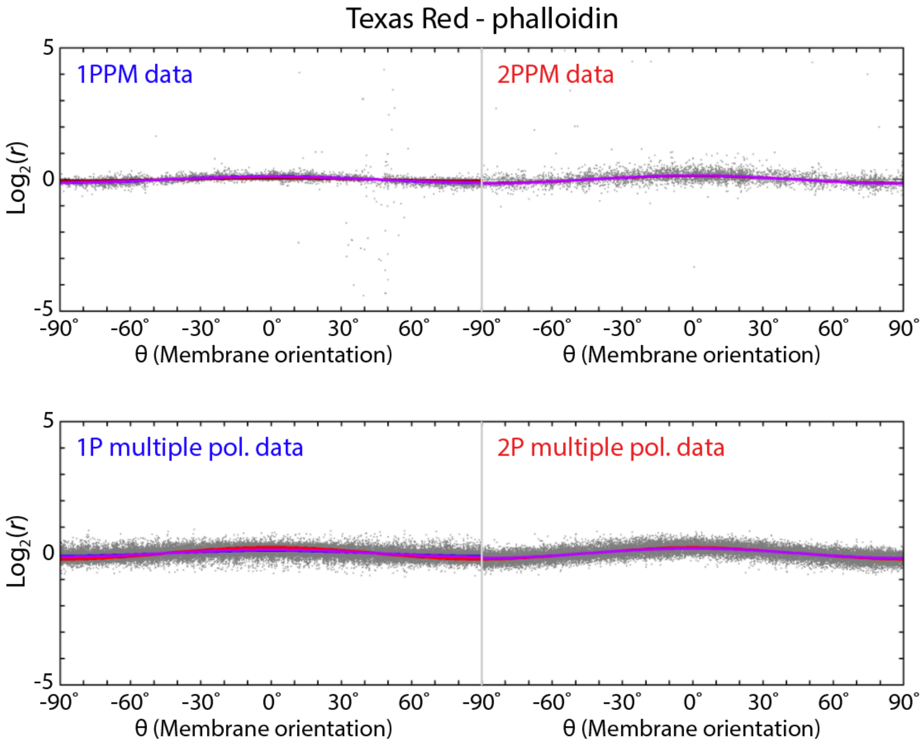

Suppl. Fig. 8: LD of microfilaments stained with Texas Red-phalloidin. Values of $\log_2(r)$, obtained from polarization microscopy observations of multiple cells, are shown as a function of filament orientation (angle θ). Top panel: data from images acquired by 1PPM and 2PPM; bottom panel: data acquired using multiple polarizations of excitation light. Blue line – fit of 1P data; dark red line – fit of 2P data; light red line – fit of 2P data with fitting parameters restricted to Gaussian distributions of molecular orientations; magenta line – fit of combined 1P and 2P data.

Suppl. Table 1

Fluorescent molecule	Sample, technique	$\log_2(r_{max})$, 1P		$\log_2(r_{max})$, 2P		α_0	σ
		mean ± 95% CI	N	mean ± 95% CI	N		
DiI	full vesicles, 1PPM/2PPM	-3.10 ± 0.02	32	-4.75 ± 0.08	25	85°	15°
	4 sections (60°), 1PPM/2PPM	-3.03 ± 0.03	31	-5.09 ± 0.16	26	81°	11°
	1 section (60°), 1PPM/2PPM	-3.20 ± 0.06	32	-5.38 ± 0.27	26	83°	11°
	1 section (15°), 1PPM/2PPM	-2.99 ± 0.12	32	-5.02 ± 0.32	26	82°	11°
clmeGFP	round cells, 1PPM/2PPM	1.33 ± 0.03	23	1.89 ± 0.05	29	26°	25°
	intact cells, 1PPM/2PPM	0.78 ± 0.05	20	0.92 ± 0.06	24	44°	15°
	intact cells, multiple pol.	0.56 ± 0.09	15	0.84 ± 0.12	16	6°	50°
	MD simulations	1.59	N/A	2.35	N/A	37°	16°
dlmeGFP	round cells, 1PPM/2PPM	2.70 ± 0.06	19	3.67 ± 0.16	33	3°	23°
	intact cells, 1PPM/2PPM	1.69 ± 0.11	19	2.16 ± 0.14	25	25°	24°
	intact cells, multiple pol.	1.96 ± 0.12	17	3.33 ± 0.20	17	25°	16°
	MD simulations	5.09	N/A	8.61	N/A	11°	8°
$G\alpha_{i1}$-L91-eYFP	round cells, 1PPM/2PPM	1.47 ± 0.05	16	2.20 ± 0.10	15	24°	24°
	intact cells, 1PPM/2PPM	0.88 ± 0.08	29	1.39 ± 0.11	11	35°	22°
	intact cells, multiple pol.	0.81 ± 0.10	25	1.50 ± 0.14	13	8°	42°
$G\alpha_{i1}$-L91-eYFP (+ NE)	round cells, 1PPM/2PPM	1.08 ± 0.06	16	1.76 ± 0.12	15	6°	38°
	intact cells, 1PPM/2PPM	0.57 ± 0.09	29	1.04 ± 0.16	11	0°	48°
	intact cells, multiple pol.	0.52 ± 0.09	18	0.83 ± 0.15	11	4°	49°
$G\alpha_o$-L91-eYFP	round cells, 1PPM/2PPM	-0.47 ± 0.05	21	-0.73 ± 0.12	15	67°	43°
	intact cells, 1PPM/2PPM	-0.16 ± 0.02	19	-0.22 ± 0.08	19	64°	59°
	intact cells, multiple pol.	-0.13 ± 0.05	21	-0.17 ± 0.08	12	50°	41°
$G\alpha_o$-L91-eYFP (+ NE)	round cells, 1PPM/2PPM	-0.20 ± 0.04	21	-0.34 ± 0.05	15	79°	61°
	intact cells, 1PPM/2PPM	0.00 ± 0.02	19	-0.01 ± 0.02	19	40°	90°
	intact cells, multiple pol.	0.01 ± 0.02	21	0.10 ± 0.11	12	37°	68°
Texas red - phalloidin	fixed cells, 1PPM/2PPM	0.12 ± 0.01	17	0.14 ± 0.02	17	43°	37°
	fixed cells, multiple pol.	0.13 ± 0.03	18	0.21 ± 0.03	14	32°	58°

Quantitative characterization of LD of the studied fluorescent molecules

Suppl. Table 2

#1 %	α_{01}, σ_1	#2 %	α_{02}, σ_2	RMSD	Entropy	$Log_2(r_{max})$, 1PPM	$Log_2(r_{max})$, 2PPM	B_{1P}	B_{2P}	C_{2P}
100 %	88°, 14°	0 %	90°, 0°	0.122	-	-2.899	-4.935	-0.7635	-1.0844	0.1577
95 %	90°, 12°	5 %	65°, 60°	0.064	3.9843	-3.016	-4.696	-0.780	-1.0948	0.1827
90 %	86°, 10°	10 %	67°, 26°	0.063	3.8171	-3.033	-4.687	-0.7822	-1.0958	0.1843
85 %	90°, 8°	15 %	59°, 4°	0.061	3.3944	-3.033	-4.710	-0.7822	-1.0953	0.1823
80 %	86°, 4°	20 %	61°, 4°	0.059	2.8052	-3.039	-4.673	-0.783	-1.0941	0.1834
75 %	86°, 0°	25 %	63°, 4°	0.058	-	-3.049	-4.679	-0.7845	-1.0948	0.1837
70 %	90°, 2°	30 %	65°, 4°	0.058	2.3200	-3.049	-4.722	-0.7844	-1.0953	0.1816
65 %	90°, 0°	35 %	67°, 6°	0.058	-	-3.028	-4.669	-0.7815	-1.0913	0.1806
60 %	88°, 2°	40 %	71°, 10°	0.059	2.7559	-3.030	-4.663	-0.7819	-1.0925	0.1823
55 %	84°, 2°	45 %	79°, 16°	0.060	3.0478	-3.022	-4.689	-0.7808	-1.0932	0.1813
50 %	74°, 12°	50 %	89°, 0°	0.060	-	-3.042	-4.656	-0.7835	-1.0938	0.1842

Results of fitting of data from LD measurements of DiI-stained GUVs by pairs of Gaussian distributions of molecular orientations. Different fractional representations of the major (50 - 100 %) and minor (0 - 50 %) Gaussian distributions were sampled and evaluated in terms of goodness of fit (RMSD) and entropy. For each fractional composition, the pair of distributions best matching the microscopy data is shown. The predicted values of $\log_2(r_{max})$ for 1PPM and 2PPM are also listed, and so are the values of the fitting equation parameters B_{1P}, B_{2P}, and C_{2P}.
