# Supplementary material for: Quantitative linear dichroism imaging of molecular processes in living cells made simple by open software tools
Source: Commun Biol. 2021 Feb 12;4:189. doi: 10.1038/s42003-021-01694-1 (PMC7881160; doi:10.1038/s42003-021-01694-1)
Supplement: Supplementary file 2 — Description of Additional Supplementary Files [file 42003_2021_1694_MOESM2_ESM.pdf]

Description of Additional Supplementary Files

File Name: Supplementary Data 1

Description: Data sets shown in plots in main figures (Fig. 3 – 9)

File Name: Supplementary Video 1

Description: A video showing a typical use of the ‘Process a polarization image (mixed image) [g]’ macro

File Name: Supplementary Video 2

Description: A video showing a typical use of the ‘Process a polarization image (multiple polarizations) [m]’ macro

File Name: Supplementary Video 3

Description: A video showing a typical use of the ‘Combine 1P, 2P data [c]’ macro

File Name: Supplementary Video 4

Description: A video showing a typical use of the ‘Fit by two Gaussian distributions [t]’ macro
