# Supplementary material for: Quantitative linear dichroism imaging of molecular processes in living cells made simple by open software tools
Source: Commun Biol. 2021 Feb 12;4:189. doi: 10.1038/s42003-021-01694-1 (PMC7881160; doi:10.1038/s42003-021-01694-1)
Supplement: Supplementary file 8 — Reporting Summary [file 42003_2021_1694_MOESM8_ESM.pdf]

nature research

Corresponding author(s): Josef Lazar

Last updated by author(s): Jan 6, 2021

Reporting Summary

Nature Research wishes to improve the reproducibility of the work that we publish. This form provides structure for consistency and transparency in reporting. For further information on Nature Research policies, see our Editorial Policies and the Editorial Policy Checklist.

Statistics

For all statistical analyses, confirm that the following items are present in the figure legend, table legend, main text, or Methods section.

n/a	Confirmed	
☐	☒	The exact sample size (*n*) for each experimental group/condition, given as a discrete number and unit of measurement
☐	☒	A statement on whether measurements were taken from distinct samples or whether the same sample was measured repeatedly
☐	☒	The statistical test(s) used AND whether they are one- or two-sided<br>*Only common tests should be described solely by name; describe more complex techniques in the Methods section.*
☒	☐	A description of all covariates tested
☐	☒	A description of any assumptions or corrections, such as tests of normality and adjustment for multiple comparisons
☐	☒	A full description of the statistical parameters including central tendency (e.g. means) or other basic estimates (e.g. regression coefficient) AND variation (e.g. standard deviation) or associated estimates of uncertainty (e.g. confidence intervals)
☐	☒	For null hypothesis testing, the test statistic (e.g. *F*, *t*, *r*) with confidence intervals, effect sizes, degrees of freedom and *P* value noted<br>*Give P values as exact values whenever suitable.*
☒	☐	For Bayesian analysis, information on the choice of priors and Markov chain Monte Carlo settings
☒	☐	For hierarchical and complex designs, identification of the appropriate level for tests and full reporting of outcomes
☐	☒	Estimates of effect sizes (e.g. Cohen's *d*, Pearson's *r*), indicating how they were calculated

Our web collection on statistics for biologists contains articles on many of the points above.

Software and code

Policy information about availability of computer code

Data collection	Microscopy images were acquired using the Fluoview FV1200 software (Olympus).
Data analysis	Images were processed in ImageJ (v1.53), using the macros (version 2.0) described in the present manuscript, available at https://github.com/jlazarlab/polarization_microscopy_macros, along with full documentation.

For manuscripts utilizing custom algorithms or software that are central to the research but not yet described in published literature, software must be made available to editors and reviewers. We strongly encourage code deposition in a community repository (e.g. GitHub). See the Nature Research guidelines for submitting code & software for further information.

Data

Policy information about availability of data

All manuscripts must include a data availability statement. This statement should provide the following information, where applicable:

- Accession codes, unique identifiers, or web links for publicly available datasets
- A list of figures that have associated raw data
- A description of any restrictions on data availability

Image processing and analysis software is freely available, along with the source code. Imaging data is available from authors upon request.

Field-specific reporting

Life sciences study design

All studies must disclose on these points even when the disclosure is negative.

Sample size	Sample size was determined as the number of individual cells or vesicles whose images were processed and quantitatively analyzed.
Data exclusions	Irregularly shaped vesicles or cells were excluded from analysis. Within a particular vesicle or a cell, sections of membrane showing irregularities (bright puncta, abrupt changes in orientation) were excluded from image processing.
Replication	Individual experiments (observations of a particular construct) were carried out in triplicates, over the course of multiple imaging sessions.
Randomization	Samples were allocated randomly into observation days.
Blinding	Image analyses of a particular sample were performed without knowledge of results of analyses of other samples.

Reporting for specific materials, systems and methods

We require information from authors about some types of materials, experimental systems and methods used in many studies. Here, indicate whether each material, system or method listed is relevant to your study. If you are not sure if a list item applies to your research, read the appropriate section before selecting a response.

Materials & experimental systems

n/a	Involved in the study
☒	☐ Antibodies
☐	☒ Eukaryotic cell lines
☒	☐ Palaeontology and archaeology
☒	☐ Animals and other organisms
☒	☐ Human research participants
☒	☐ Clinical data
☒	☐ Dual use research of concern

Methods

n/a	Involved in the study
☒	☐ ChIP-seq
☒	☐ Flow cytometry
☒	☐ MRI-based neuroimaging

Eukaryotic cell lines

Policy information about cell lines

Cell line source(s)	HEK 293 cells were purchased as such from ATCC. Fixed BPAE cells were purchased as such from ThermoFisher
Authentication	Cell lines were used without authentication.
Mycoplasma contamination	Cell lines were tested for mycoplasma contamination every 6 months, with negative results.
Commonly misidentified lines (See ICLAC register)	-
